# Electro-Acupuncture Ameliorated MPTP-Induced Parkinsonism in Mice via TrkB Neurotrophic Signaling

**DOI:** 10.3389/fnins.2019.00496

**Published:** 2019-05-14

**Authors:** Yingke Zhao, Dan Luo, Zhipeng Ning, Jianhui Rong, Lixing Lao

**Affiliations:** School of Chinese Medicine, Li Ka Shing Faculty of Medicine, The University of Hong Kong, Pok Fu Lam, Hong Kong

**Keywords:** Parkinson’s disease, electro-acupuncture, brain-derived neurotrophic factor, TrkB, MPTP

## Abstract

Neurotrophins, such as brain-derived neurotrophic factor (BDNF), have shown promise as neuroprotective agents, indicating their potential in therapeutic strategies for neurodegenerative disease. However, the inherent bioactivity and pharmaceutical limitations of BDNF compromise its clinical efficacy. Research has documented the beneficial effects of electroacupuncture (EA) against neurodegeneration, possibly by BDNF-mediated mechanisms. The present study was designed to clarify whether EA can mount a neuroprotective effect in mice lesioned with MPTP (1-methyl-4-phenyl-1,2,3,6-tetrahydropyridine) via stimulation of the BDNF-TrkB signaling pathway. We found that EA not only ameliorated the motor dysfunction but also restored the dopaminergic neuronal function and upregulated BDNF expression in MPTP-lesioned mice. Interestingly, the TrkB inhibitor K252a abolished the neuroprotective effects of EA. Western blot analyses further demonstrated that EA might recover the level of phospho-Akt, phospho-ERK1/2, and BDNF against MPTP neurotoxicity via reversing the imbalance between TrkB FL and TrkB T1. Taken together, the results of the present study show that EA stimulation can ameliorate MPTP-induced parkinsonism in mice. Such a neuroprotective effect may be partially mediated via restoring TrkB neurotrophic signaling.

## Introduction

Parkinson’s disease (PD) is characterized by the progressive loss of dopaminergic neurons in the substantia nigra par compacta (SNpc) and by impairments in motor function as well as other autonomic nervous system activities (Dexter and Jenner, 2013; Kalia and Lang, 2016). Although the exact etiology of PD remains elusive, recent studies have revealed that the neuronal functions may be impaired by several risk factors including oxidative stress, neuroinflammation, glutaminergic toxicity, protein misfolding and aggregation, and lack of neurotrophins (Kalia and Lang, 2015). Under oxidative stress, the physiological functions of neurotrophins [e.g., nerve growth factor and brain-derived neurotrophic factor (BDNF)] and the related tyrosine kinase receptors (Trks) are affected, subsequently jeopardizing the survival, differentiation, and patterning of neurons (Yamada et al., 2001; Bruno and Cuello, 2012). Disruption of BDNF biosynthesis failed to support dopaminergic neurons, whereas BDNF polymorphisms are associated with a susceptibility to PD (Hong et al., 2003). Previous studies demonstrated that exogenous BDNF could prevent the neurotoxicity of 1-methyl-4-phenyl-1,2,3,6-tetrahydropyridine (MPTP) and 6-hydroxydopamine (6-OHDA) in dopaminergic neurons (Frim et al., 1994; Levivier et al., 1995). Interestingly, ectopic overexpression of BDNF ameliorated the motor dysfunction in 6-OHDA-treated animals (Klein et al., 1999). These studies indicate that BDNF is important for preserving the dopaminergic neurons and preventing or retarding neurodegeneration. Moreover, tyrosine kinase receptor type B (TrkB) is well known to mediate the neurotrophic function of BDNF. The TrkB gene encodes different isoforms of the TrkB receptor: full-length (TrkB FL) and splicing truncated isoforms (TrkB T1 or T2) (Otani et al., 2017). Notably, the truncated isoform TrkB T1 is regarded as the dominant negative form and may thereby suppress the neurotrophic activity of BDNF (Gupta et al., 2013). TrkB T1 is upregulated in situations such as stress, hypoxia, excitotoxicity, and neurodegeneration (Ferrer et al., 1999; Vidaurre et al., 2012). Collectively, neuroprotective therapy is needed to recover the balance of TrkB FL and TrkB T1 (Andero et al., 2014).

Acupuncture and electroacupuncture (EA) are well documented for their beneficial effects against various neurological disorders (Ghaffari and Kluger, 2014; Solanki et al., 2016). Accumulating evidence from clinical studies also provides support for the beneficial effect of EA in the management of PD, and clinicians have emphasized its application as a complementary strategy for treating the non-motor symptoms of PD (Jiang et al., 2018). As a non-pharmacological approach, EA has been investigated by several groups for its biological effects against PD (Xiao et al., 2018). EA was found to regulate the levels of various neurotransmitters such as dopamine, glutamate, and acetylcholine (Rui et al., 2013; Li et al., 2017). EA exhibited potent antioxidant properties in PD models, in particular by activating the nuclear factor E2–related factor 2 (Nrf2) signaling pathway (Lv et al., 2015). EA might elicit anti-neuroinflammatory activity through suppressing glial activation (Deng et al., 2015). As for the effects of EA on neurotrophic factors (e.g., BDNF), Liang et al. (2002) observed that EA upregulated the expression of BDNF in 6-OHDA-lesioned rats. Kim et al. (2011) also observed that manual acupuncture intervention could induce the activation of the PI3K/Akt pathway. Interestingly, Lin reported that EA enhanced the activation of BDNF and the phosphorylation of downstream signaling molecules such as Akt and ERK in MPP^+^-induced rat models (Lin et al., 2017). These studies suggest the potential of EA in the activation of the BDNF signaling pathway.

In the present study, we propose to further identify the effects of EA on the BDNF-TrkB signaling pathway as a neuroprotective mechanism against MPTP neurotoxicity. We focus on the expression level and the post-translational processing of TrkB within the context of MPTP-induced neurodegeneration.

## Materials and Methods

### Experimental Design

**Figure 1:**
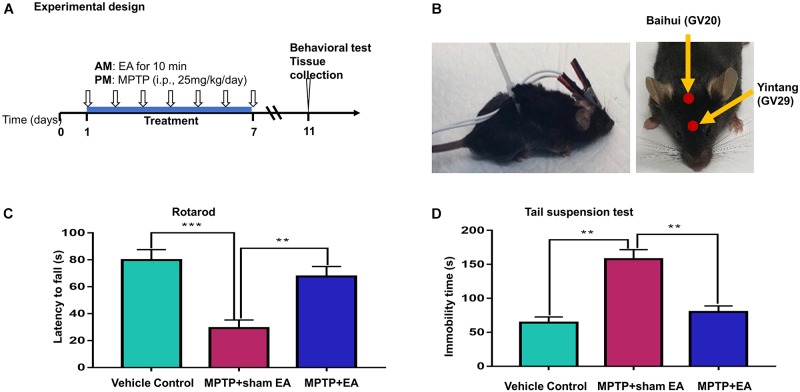
EA ameliorated the deficit of motor functions in MPTP-lesioned mouse model **(A)** Experimental design. Mice were randomly divided into three groups: vehicle control, MPTP+sham EA, MPTP+EA. Mice received MPTP (25 mg/kg/day) for 7 consecutive days. EA treatment was carried out at 4 h before MPTP injection. After one-week treatment, mice were kept for 3 days and assessed by behavioral test and biochemical analysis. **(B)** Diagram for EA stimulation. EA was applied at GV20 and GV29. Needles were inserted into acupoint at 3 mm under the skin. The electrodes were connected to the needles and secured with a plastic wire. EA was carried out for 10 min per session. **(C)** Rotarod test performance to assess the behavior impairments. The on-rotarod time for mice was recorded. **(D)** Tail suspension test. The immobility time for mice was recorded over 6 min. The results were presented as mean ± SEM from 3 independent experiments (*n* = 10), ^∗∗^*p* < 0.01, ^∗∗∗^*p* < 0.001.

The design of our animal experiments is outlined in Figure 1A. To observe the effect of EA on MPTP-triggered parkinsonism symptoms, 30 mice were randomly divided into three groups: vehicle control group, model group (MPTP + sham EA), and EA group (MPTP + EA). Mice in the model and EA groups received MPTP (25 mg/kg/day) via intraperitoneal injection every afternoon for 7 consecutive days, whereas animals in the vehicle control group received the same volume of saline. The EA treatment was carried out every morning, 4 h prior to the MPTP injection, for 7 days, whereas mice from the control group and the model group were subjected to sham EA treatment. The detailed procedures for EA and sham EA are described below. In the next step, to identify the role of the TrkB receptor in the protective effect of EA, we randomly divided the mice in three groups (10 mice/group): model group (MPTP+ sham EA), EA group (MPTP + EA), and K252a group (MPTP + EA + K252a). Prior to EA treatment, mice in the K252a group were pre-treated with K252a (a TrkB inhibitor) via intraperitoneal injection (5 μg/kg/day) 1 h in advance, whereas mice in the other two groups received the equivalent volume of vehicle (saline). Mice from the EA group and the K252a group were treated by EA every morning for 7 days, whereas mice from MPTP group were subjected to sham EA.

### Biochemical Reagents

Antibody against tyrosine hydroxylase (TH) was purchased from Merck Millipore (Billerica, MA, United States). Polyclonal rabbit anti-BDNF antibody was purchased from Santa Cruz Biotechnology Inc., (Santa Cruz, CA, United States). Antibodies against TrkB, ERK, phospho-ERK, AKT, phospho-AKT, CREB, phospho-CREB, GAPDH, and Alexa Fluor 594-conjugate anti-rabbit IgG antibody were obtained from Cell Signaling Technology (Boston, MA, United States). Protein assay dye reagent concentrate was purchased from Bio-Rad (Hercules, CA, United States). Enhanced chemiluminescence detection reagents were obtained from GE Healthcare (Uppsala, Sweden). A 3,3-*N*-diaminobenzidine tetrahydrochloride (DAB) substrate kit was purchased from Dako Corporation (Carpintera, CA, United States). Anti-rabbit horseradish peroxidase (HRP)-conjugated IgG secondary antibody and MPTP were purchased from Sigma-Aldrich (St. Louis, MO, United States). Mouse anti-BDNF antibody and proteinase inhibitor K252a were bought from Abcam (Cambridge, United Kingdom). Goat-anti Mouse Alexa-568 was obtained from Invitrogen (Carlsbad, CA, United States).

### Animal Husbandry and MPTP-Induced PD Model

Adult C57BL/6N male mice (6–8 weeks, weight 21–25 g) were supplied by the Laboratory Animal Unit, University of Hong Kong. The mice were housed in a 12-h/12-h light/dark cycle under the conditions of constant temperature and humidity. All the experimental procedures were performed according to the regulations of the Hong Kong University Committee on the Use of Live Animals in Teaching and Research (CULATR No. 4196-16). The method of establishing MPTP-induced parkinsonism mice followed our previous work (Zhao et al., 2015). Mice received MPTP (25 mg/kg/day) via intraperitoneal injection every afternoon for 7 consecutive days, whereas animals in the vehicle control group received the same volume of saline. After the last injection of MPTP, mice were kept in isolation for 3 days for potential neurotoxin risk. Finally, mice were moved out, assessed for behavioral performance, and subsequently sacrificed after the 3-day isolation.

### EA and Sham EA Procedure

Electroacupuncture treatment was performed at the time point of 4 h prior to daily MPTP injection for 7 consecutive days essentially as previously described (Lao et al., 2004). The acupoints (i.e., GV20, Baihui; GV29, Yintang) were selected in our experiments (shown in Figure 1B). The acupoint GV20 is located in the vertex on the midline (5 *cun* posterior to the anterior hairline), whereas the acupoint GV29 is located midway between the medial ends of the eyebrows in humans. The equivalent position in mice was selected to mimic these two acupoints (Yin et al., 2008). Before the experiment, mice had the fur over the acupoints removed and the exposed skin cleaned. During the treatment, disposable intradermal acupunctures (0.22^∗^5 mm, Hwato, China) were inserted into the acupoints to a depth of 3 mm. Electrodes were then connected to the end of each needle; the needles and the electrodes were stabilized by wiring the mouse body. EA stimulation was subsequently performed using an ES-160, six-channel programmable EA stimulator (ITO physiotherapy & rehabilitation Co., Tokyo, Japan), with intensity 1.5 mA, frequency 2 Hz, and pulse width 100 μs for 10 min. During EA stimulation, mice were placed in a plastic chamber without restraint other than a plastic wire around the body to stabilize the electrical wire. Animals from the control and MPTP groups received a sham EA procedure. Briefly, needles were taped onto the surface at the two acupoints, and the electrodes were connected to the ends of the needles, but no electricity stimulation was performed. The same wire stabilization procedure and similar plastic chamber were used during the treatment, to minimize potential confounders.

### Behavioral Test

#### Rotarod Performance Test

Mice were assessed for MPTP-triggered parkinsonism impairments by a rotarod performance test (Harvard apparatus, Holliston, MA, United States). Mice were isolated for 3 days after the last MPTP administration prior to the test. On the day of the behavioral test, mice were first pre-trained on the machine three times for 3 min each. After resting for 2 h, mice were placed on the rotating horizontal rod (constant 15 rpm) for 5 min. The time for each mouse staying on the rod was automatically recorded by the rotarod apparatus. Each mouse was tested for three times, and after each test the mice were put back to their cage for a 30-min intertrial interval.

#### Tail Suspension Test

The tail suspension test, was conducted 4 h after the rotarod test as previously described (Luo et al., 2018). Briefly, mice were suspended from a bar by adhering a piece of tape to the tail and attaching the other end to the bar. The distance between the mouse’s nose and the apparatus was kept to about 20–25 cm. The status of each mouse during a 6-min hanging session was tracked by video; the observers thereafter analyzed the video and recorded the agitation and immobility times of each mouse during the test.

### Western Blot Analysis

The expression levels of BDNF, TrkB, and other related proteins were detected by Western blotting as described elsewhere (Zhao et al., 2017). Midbrain tissues were isolated and lysed in RIPA buffer containing a protease inhibitor cocktail. The proteins were recovered by centrifugation at 13,000 rpm for 15 min at 4°C. The protein concentration was determined with protein assay dye reagent. Proteins (20 μg) were resolved on 10% SDS–polyacrylamide gels and transferred onto a PVDF membrane. After 2 h incubation in Tris buffer with 1% Tween-20 and 5% BSA, the membranes were probed with primary antibodies overnight at 4°C, and subsequently detected with goat anti-rabbit IgG-HRP-conjugated secondary antibodies for another 3 h at 4°C. The blots were visualized with ECL under a Bio-Rad GelDoc imaging system (Hercules, CA, United States). The gel images were analyzed by Image Lab^TM^ software 5.1.

### Immunohistochemistry

The expression level of TH in the midbrain was detected by immunohistochemical staining as previously described (Zhao et al., 2015). After the behavioral tests, mice were transcardially perfused with saline and 4% paraformaldehyde under anesthetic conditions. The brain tissues were collected and post-fixed in 4% paraformaldehyde overnight at 4°C. The samples were then immersed in 4% paraformaldehyde solution containing 30% sucrose overnight until the tissues sank, for cryoprotection. Tissues were fixed and embedded in Tissue-Tek O.C.T. Compound (Sakura, United States) and stored at –80°C. The cryosections were cut into serial coronal sections with a thickness of 30 μm on a freezing microtome (Model CM-1850, Leica, Germany). After thawing at room temperature for 1 h, the cryostat sections were heated in antigen retrieval citrate buffer for 30 min. After thawing at room temperature for 1 h, the cryostat sections were heated in antigen retrieval citrate buffer for 30 min. The slides were cooled, immersed in 0.3% hydrogen peroxide solution to block endogenous peroxidase, and sequentially blocked with 5% goat serum and 0.5% Triton X-100 in PBS for 30 min at room temperature. The slides were drained on tissue paper, then incubated with anti-TH primary antibody overnight at 4°C. Following several washes, the bound antibodies were detected with HRP-conjugated goat anti-rabbit secondary antibody for 2 h at room temperature. The HRP was then assayed with a DAB substrate kit from Dako (Carpintera, CA, United States). The slices were counterstained with hematoxylin, then observed under an Olympus microscope (Olympus Corp, Tokyo, Japan). The number of TH^+^ neurons of each animal was counted within three non-overlapping areas at 10× magnification.

### Immunofluorescence Staining

The expression of BDNF in the SNpc was examined by immunofluorescence staining. The cryosections were prepared as described for immunohistochemical analysis. After antigen retrieval, the slides were sequentially immersed in 0.5% Triton X-100 in PBS for 30 min, and then blocked in 5% goat serum for 2 h. The sections were probed with antibody against BDNF overnight at 4°C, washed three times with PBS, and detected with Alexa Fluor 568-conjugated goat anti-mouse IgG secondary antibody. The cell nuclei were stained with 4’-6-diamidino-2-phenylindole for 10 min. Prior to the examination of fluorescence, the slides were mounted with coverslips in mounting medium. The images were then acquired under a Zeiss LSM 780 confocal microscope (Carl-Zeiss, Jena, Germany). The positive cells were enumerated from three different views in each specimen.

### Statistical Analysis

Behavioral assessments were represented as means ± SEM, whereas other experiments were expressed in mean ± SD. The difference was analyzed by one-way ANOVA, followed by Dunnett’s *post hoc*, using GraphPad Prism software 7.00 (La Jolla, CA, United States). A value of *p* < 0.05 was considered statistically significant.

## Results

### EA Ameliorated the Deficit of Motor Functions in an MPTP-Lesioned Mouse Model

To assess the effects of EA on the neuronal impairments, mice were treated with MPTP for 7 consecutive days and evaluated by rotarod test and tail suspension test. The time of latency in the rotarod performance test was recorded to indicate motor dysfunction, whereas the immobility time in the tail suspension test was measured for depressive behaviors (Taylor et al., 2010). As shown in Figure 1C,D, the rotarod performance test showed that MPTP shortened the time that mice remained on the rotarod (29.3±6.029 s (MPTP + sham EA) vs. 79.8±7.791 s (vehicle control), *n* = 10, *p* = 0.0001). EA treatment effectively prolonged the time on the rotarod for mice compared with the MPTP group (67.7±7.336 s (MPTP + EA) vs. 29.3±6.029 s (MPTP + sham EA), *n* = 10, *p* = 0.0014). The tail suspension test showed that MPTP increased the immobility time of mice compared with vehicle control mice (157.8±13.91 s (MPTP + sham EA) vs. 64.2±8.346 s (vehicle control), *n* = 10, *p* = 0.001). EA treatment successfully reduced the immobility time for MPTP-lesioned mice (79.9±8.877 s (MPTP + EA) vs. 157.8±13.91 s (MPTP + sham EA), *n* = 10, *p* = 0.001). Results from both the rotarod test and tail suspension test showed that the 7-day injection of MPTP triggered a severe motor disability in mice, whereas the EA treatment dramatically ameliorated the impairment observed in both the rotarod performance and tail suspension tests.

### EA Reduced the Loss of Dopaminergic Neurons in MPTP-Treated Mice

**Figure 2:**
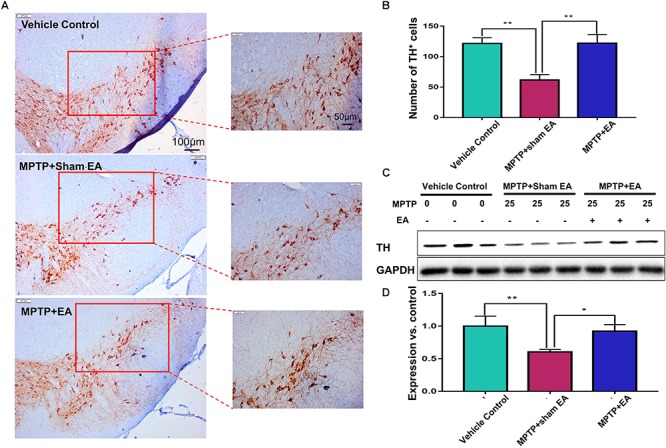
EA reduced the loss of dopaminergic neurons in MPTP-treated mice After behavioral test, midbrain tissues were collected and analyzed by immunohistochemical analysis. The number of TH^+^ cells on each slide was counted from three different views at 10× magnification under an Olympus microscope. **(A)** Representative images (with 10× and 20× magnification) were shown. Scale bar represented 100 μm, 50 μm in length, respectively. **(B)** The statistical analysis for the expression of TH^+^ cells among each group. The results were presented as mean ± SD (*n* = 4). ^∗^*p* < 0.05, ^∗∗^*p* < 0.01. **(C)** TH expression determined by immunoblot. Midbrain tissues were lysed and analyzed by Western blotting with anti-TH and GAPDH (as loading control). Representative blots from three mice of each group were shown. **(D)** Quantification for the immunoblots. The blots were quantified by a densitometric method by ImageLab 5.1 (Bio-Rad). ^∗^*p* < 0.05, ^∗∗^*p* < 0.01.

The key pathological change of PD is dopaminergic neuron loss in the SNpc; therefore, we observed the loss of dopaminergic neurons by detecting the expression of TH. Immunohistochemical analysis and Western blotting were used in this regard. As shown in Figure 2A,B, MPTP downregulated TH expression compared with vehicle controls (*n* = 4, *p* = 0.0012). EA treatment restored the level of TH expression [121.9±14.46 (MPTP + EA) vs. 61.75±8.757 (MPTP + sham EA), *n* = 4, *p* = 0.0010]. Western blot analysis also confirmed that EA effectively restored TH expression in the midbrain compared with MPTP without EA [0.92±0.101 (MPTP + EA) vs. 0.61±0.035 (MPTP + sham EA), *n* = 3, *p* = 0.02] (Figure 2C,D). Thus, our results validated that EA treatment not only preserved the expression of TH in the midbrain but also effectively protected the dopaminergic neurons from MPTP neurotoxicity.

### EA Preserved the Functions of the BDNF-TrkB Signaling Pathway

**Figure 3:**
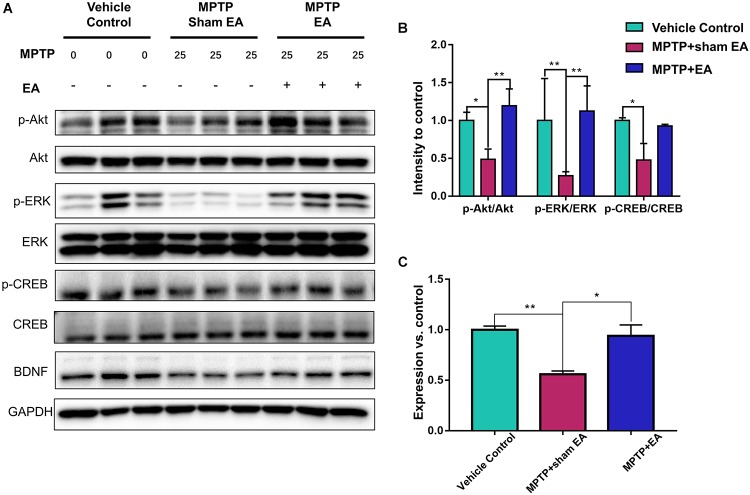
EA preserved the functions of BDNF-TrkB signaling pathway. The midbrain tissues were lysed and analyzed by Western blotting using antibodies against Akt, phospho-Akt, ERK1/2, phospho-ERK1/2, CREB, phospho-CREB, and BDNF, GAPDH serves as loading control. **(A)** Representative blot was presented. **(B)** The quantitative analysis for the phosphorylation of Akt, ERK1/2, and CREB. **(C)** Quantification of the BDNF expression detected by western blot. The signal intensities of protein bands (*n* = 3) were determined by a densitometric method, and quantitatively analyzed by one-way ANOVA. ^∗^*p* < 0.05, ^∗∗^*p* < 0.01.

**Figure 4:**
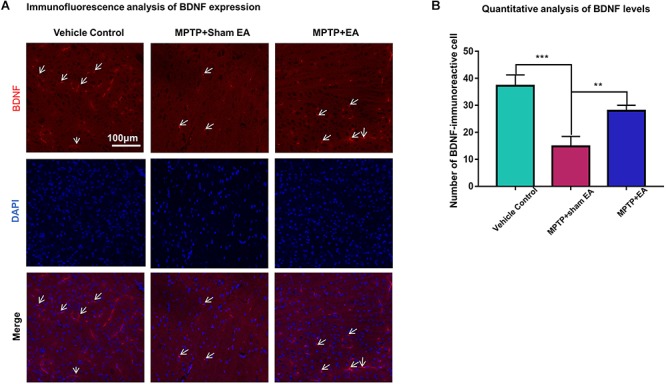
EA reversed the expression of BDNF against MPTP neurotoxicity. Midbrain cryosections were incubated with primary antibody against BDNF and Alexa Fluor 596-conjugated secondary antibody. The cell nuclei were stained with DAPI for 10 min. The SN areas were examined under a Zeiss LSM 780 confocal microscopy. **(A)** Representative images with 20× amplification were shown. Scale bar represents 100 μm in length. **(B)** Quantitative analysis of BDNF expression detected by immunostaining. Numbers of BDNF-immunoreactive cells in the SN region was quantified and expressed in mean ± SD (*n* = 3). ^∗∗^*p* < 0.01, ^∗∗∗^*p* < 0.001.

To observe the effect of EA on the BDNF-TrkB signaling pathway, we examined the expression levels of BDNF and the activation of its downstream signaling cascades, such as phospho-Akt, Akt, phospho-ERK1/2, ERK1/2, phospho-CREB, and CREB, by Western blotting with specific antibodies. As illustrated in Figure 3A,C, MPTP decreased BDNF expression compared with vehicle control [0.56±0.056 (MPTP + sham EA) vs. 1±0.064 (vehicle control), *n* = 3, *p* = 0.0069], whereas EA treatment significantly recovered the level of BDNF expression [0.94±0.19 (MPTP + EA) vs. 0.56±0.056 (MPTP + sham EA), *n* = 3, *p* = 0.0136]. The activation of downstream signaling cascades was also investigated by Western blot analysis (Figure 3A,B). After a 7-day administration, MPTP largely inactivated the survival signaling transduction, as indicated by the weaker intensity of the phosphorylated bands in each sample compared with the vehicle control group: phospho-AKT/AKT (*n* = 3, *p* = 0.0373), phospho-ERK/ERK (*n* = 3, *p* = 0.0038), and phospho-CREB/CREB (*n* = 3, *p* = 0.0339). Interestingly, EA stimulation effectively increased the phosphorylation levels of phospho-AKT (*p* = 0.0048), phospho-ERK (*p* = 0.0010), but did not affect phospho-CREB (*p* = 0,0695), compared with that in MPTP-treated animals. BDNF expression in the SN was also verified by immunofluorescence analysis. Figure 4A is the representative images of immunofluorescence; as expected, MPTP treatment reduced BDNF-immunoreactive signals in the SNpc [14.8±3.72 (MPTP + sham EA) vs. 37.2±4.02 (vehicle control), *n* = 3, *p* = 0.0003] (Figure 4B). Interestingly, EA stimulation elevated the number of BDNF^+^ cells against MPTP-lesioned group [28±2 (MPTP + EA) vs. 14.8±3.72 (MPTP + sham EA), *n* = 3, *p* = 0.0053].

### EA Recovered MPTP-Induced Disruption to the Ratio of TrkB FL vs. TrkB T1

**Figure 5:**
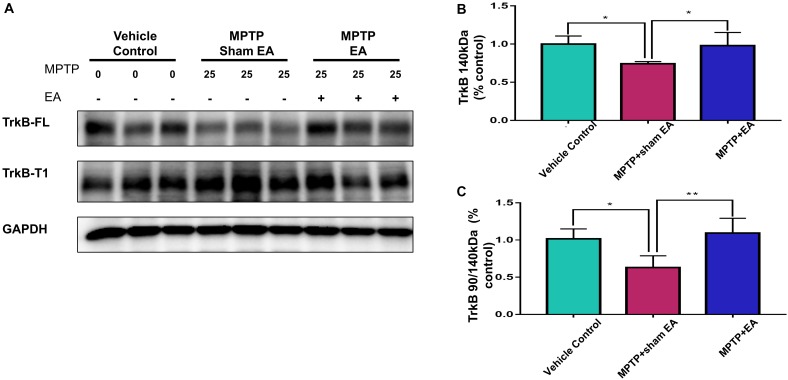
EA recovered MPTP-induced disruption to the ratio of TrkB FL vs. TrkB T1. The mesencephalon proteins were extracted and analyzed by Western blotting for the expression of TrkB FL (140 kDa) and TrkB T1 (90 kDa). GAPDH serves as loading control. **(A)** Representative blots were shown. **(B)** Quantitative analysis for the expression of TrkB FL (140 kDa). **(C)** Ratio of TrkB T1/TrkB FL determined by western blot. The intensity of each band was determined by a densitometric approach. Quantitative analysis (*n* = 4) were conducted by one-way ANOVA. ^∗^*p* < 0.05, ^∗∗^*p* < 0.01.

To clarify how EA affected TrkB receptors, we next analyzed the levels of two TrkB receptor isoforms by Western blotting. As shown in Figure 5A,B, MPTP reduced the level of TrkB FL over the 7-day stimulation compared with vehicle [0.74±0.029 (MPTP + sham EA) vs. 1±0.106 (vehicle control), *n* = 4, *p* = 0.0232]. In contrast, MPTP increased the level of truncated TrkB T1 (Figure 5A). Moreover, MPTP eventually disrupted the TrkB FL/TrkB T1 ratio [0.63±0.159 (MPTP + sham EA) vs. 1.02±0.135 (vehicle control), *n* = 4, *p* = 0.0176, Figure 5C]. Importantly, EA stimulation upregulated the expression of functional TrkB FL [0.98±0.172 (MPTP + EA) vs. 0.74±0.029 (MPTP + sham EA), *n* = 4, *p* = 0.0340], giving rise to the recovery of the MPTP-disrupted TrkB FL/TrkB T1 ratio [1.09±0.20 (MPTP + EA) vs. 0.63±0.159 (MPTP + sham EA), *n* = 4, *p* = 0.0064].

### The Protective Effect of EA Can Be Abrogated With the TrkB Inhibitor K252a

**Figure 6:**
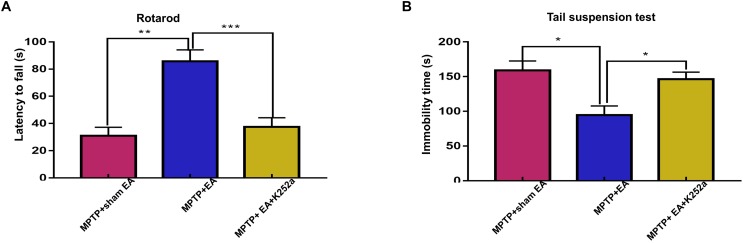
The administration of TrkB inhibitor K252a effectively abolished the effect of EA on MPTP impaired the motor function. 1 h prior to each EA session, K252a (5 μg/kg/day) or vehicle was administered to mice via i.p. injection. **(A)** The effect of K252a on rotarod performance test. The rodding time of each mice were analyzed. **(B)** The behavioral deficits were assessed by tail suspension test. The immobility time of each mice within 6-min were recorded. The results were presented as mean ± SEM from 3 independent experiments (*n* = 10). ^∗^*p* < 0.05, ^∗∗^*p* < 0.01, ^∗∗∗^*p* < 0.001.

**Figure 7:**
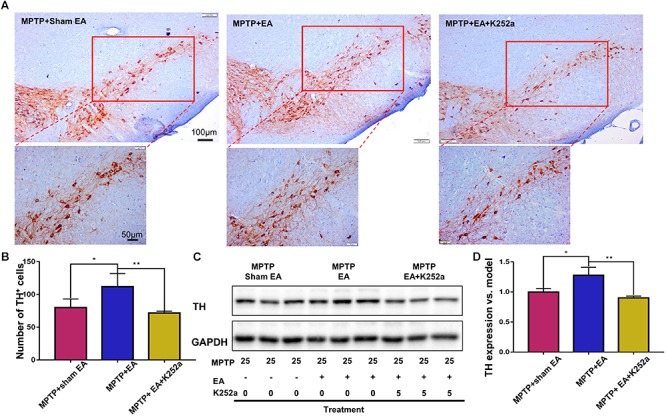
EA preserved dopaminergic neuron survival can be abrogated by K252a. The brain cryosections were sequentially incubated with TH antibody, then visualized by DAB substrate kit. **(A)** The represented photos from each group were shown (with 10× and 20× magnification), scale bar represented 100 μm, 50 μm in length, respectively. **(B)** Statistical analysis for the number of TH^+^ cells from each group. The numbers of TH^+^ cells of each slide were counted for three non-overlapping areas at 10× magnification via an Olympus microscope. The results were presented as mean ± SD of three independent experiments. **(C)** Midbrain protein were extracted and probed with anti-TH and GAPDH (as loading control) via western blotting technique. The representative blots were shown. **(D)** The quantification for TH expression probed by western blot. The blots were quantified in a densitometric method by ImageLab 5.1 (Bio-Rad). ^∗^*p* < 0.05, ^∗∗^*p* < 0.01.

**Figure 8:**
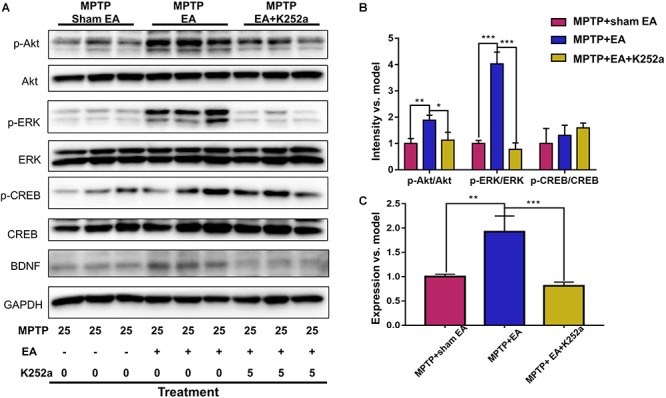
K252a administration can effectively inhibited EA enhanced BDNF signaling pathway transduction. **(A)** The midbrain tissues were lysed and analyzed by Western blotting using antibodies against Akt, phospho-Akt, ERK1/2, phospho-ERK1/2, CREB, phospho-CREB, and BDNF. Representative blot was shown. GAPDH serves as loading control. **(B)** Statistical analysis for the phosphorylation of Akt, ERK1/2, and CREB. **(C)** Quantitative analysis for the expression level of BDNF. The signal intensities of protein bands (*n* = 3) were determined by a densitometric method, and quantitatively analyzed by one-way ANOVA. ^∗^*p* < 0.05, ^∗∗^*p* < 0.01, ^∗∗∗^*p* < 0.001.

To further confirm the involvement of TrkB in the EA-regulated BDNF elevation, we used the TrkB inhibitor K252a to observe whether it could abrogate the effect of EA. Briefly, 1 h before the EA treatment, the mice in the inhibitor group received an additional injection of K252a (5 μg/kg/day, i.p.). The protective effects of EA (i.e., behavioral change, dopaminergic neuron survival, and BDNF expression) were determined. As shown in Figure 6A, K252a abrogated the EA effect, as seen by a reduction in on-rotarod time [37.5±6.718 s (MPTP + EA + K252a) vs. 85.8±8.358 s (MPTP + EA), *p* = 0.0007], resulting in a disruption comparable to that of the MPTP group [37.5±6.718 s (MPTP + EA + K252a) vs. 31±6.206 s (MPTP + sham EA), *p* = 0.7474]. In the tail suspension test, the K252a group prolonged the EA-improved immobility time [146.4±9.976 s (MPTP + EA + K252a) vs. 94.6±13.15 s (MPTP + EA), *p* = 0.0451], showing an immobility time similar to that seen in the MPTP group [146.4±9.976 s (MPTP + EA + K252a) vs. 158.9±13.66 s (MPTP + sham EA), *p* = 0.6998, Figure 6B]. The effect of EA on preserving the dopaminergic neurons was also compromised with the K252a injection. The immunohistochemical analysis of TH expression showed a level comparable to MPTP [71.67±2.764 (MPTP + EA + K252a) vs. 112.1±19.7 (MPTP + EA), *p* = 0.0046; 71.67±2.764 (MPTP + EA + K252a) vs. 79.92±13.09 (MPTP + sham EA), *p* = 0.6208, Figure 7A,B]; results from the Western blot analysis also showed a trend similar to that obtained with immunohistochemistry [0.9±0.03 (MPTP + EA + K252a) vs. 1.3±0.13 (MPTP + EA), *p* = 0.0029, 0.9±0.03 (MPTP + EA + K252a) vs. 1±0.058 (MPTP + sham EA), *p* = 0.3333, Figure 7C,D]. Moreover, the additional utilization of K252a successfully blocked the effect of EA on BDNF expression, as was detected by the Western blot shown in Figure 8 [0.81±0.076 (MPTP + EA + K252a) vs. 1.9±0.32 (MPTP+ EA), *p* = 0.0008, 0.81±0.076 (MPTP + EA + K252a) vs. 1±0.049 (MPTP + sham EA), *p* = 0.4359]. The activation of the BDNF downstream signaling cascades that was enhanced by EA stimulation was also jeopardized with K252a administration, as shown by a marked decrease in intensity of the phosphorylation of Akt and ERK1/2, but not CREB expression, as compared with the EA-treated group {p-AKT/AKT [*p* = 0.0204 (MPTP + EA + K252a vs. MPTP+ EA), *p* = 0.8555 (MPTP + EA + K252a vs. MPTP+ Sham EA)], p-ERK/ERK [*p* = 0.0001 (MPTP + EA + K252a vs. MPTP+ EA), *p* = 0.6219 (MPTP + EA + K252a vs. MPTP+ Sham EA)], and p-CREB/CREB [*p* = 0.4752 (MPTP + EA + K252a vs. MPTP+ EA), *p* = 0.0732 (MPTP + EA + K252a vs. MPTP+ Sham EA)], Figure 8A,B}.

## Discussion

The disruption of the BDNF-TrkB signaling pathway is implicated in neurodegenerative diseases (Andero et al., 2014). The potential of BDNF-based therapy is hindered by the dose and delivery in translational studies (Nagahara and Tuszynski, 2011). Acupuncture and EA may have potential for treatment to retard or reverse neurodegeneration in PD (Eng et al., 2006; Lei et al., 2016; Kong et al., 2018). Earlier studies suggested a role for BDNF in EA-induced neuroprotection. In the present study, we not only validated the effect of EA on BDNF expression against MPTP neurotoxicity but also determined the effects of EA on the TrkB FL/TrkB T1 ratio and the activation of downstream signals, such as PI3K/Akt and ERK1/2.

Electroacupuncture combines the traditional effects of acupuncture and electrical stimulation for potential neuroprotective properties against parkinsonism symptoms. The earlier study by Liang et al. (2002) reported that EA stimulation on GV20 and GV14 could upregulate the expression of BDNF in 6-OHDA-lesioned rats. Kim et al. (2011) applied manual acupuncture at GB34 to activate the neuroprotective PI3K/Akt pathway. Along the same line of evidence, Lin et al. found that EA stimulation on GB34 and LR3 not only enhanced BDNF expression but also induced the activation of the signaling proteins Akt and ERK in MPP^+^-induced rat models (Lin et al., 2017). Acupoints such as GB34 (Yang Ling Quan), ST36 (Zu San Li) and GV20 (Bai Hui) are frequently selected to achieve neuroprotective properties. GV20 and GV29 are used as scalp acupuncture for neurological disorders (Lee et al., 2013). More experiments are called for to verify the effectiveness of scalp acupuncture for PD. The present study was designed to observe the therapeutic effect of scalp acupuncture in conscious animals. EA stimulation was performed in mice without restraints. To avoid the disruption of electrical stimulation, we selected two acupoints (GV20 and GV29) on the head.

Brain-derived neurotrophic factor regulates the survival and activity of dopaminergic, motor, and sensory neurons (Hyungju and Mu-Ming, 2012). The reduction of BDNF expression in the striatum and serum was detected in PD patients (Mogi et al., 1999; Scalzo et al., 2010). Nevertheless, the ectopic overexpression of BDNF could protect the neurons against 6-OHDA-induced neurotoxicity in mice (Levivier et al., 1995). Tsukahara et al. also demonstrated the efficacy of BDNF against MPTP-induced damage in non-human primates (Tsukahara et al., 1995). It was previously shown that acupuncture reduced parkinsonism symptoms, rescued dopaminergic neurons, and restored the expression of dopamine transporters against MPTP-induced neurotoxicity (Choi et al., 2011). Moreover, several studies revealed that EA could ameliorate MPTP-induced neurotoxicity by upregulating BDNF, GDNF, and cyclophilin A, thereby ameliorating the pathological changes in PD (Chen et al., 2007; Jeon et al., 2008; Joh et al., 2010). The present study corroborated that EA could alleviate motor impairments and enhance the survival of dopaminergic neurons against MPTP neurotoxicity. Western blotting and immunofluorescence analysis further verified the effects of EA stimulation on BDNF expression in the SNpc against MPTP-induced disruption. Moreover, apart from the observation of the effect of EA on BDNF expression, we additionally determined the role of the TrkB receptor in EA-regulated neurosurvival.

It is well known that BDNF regulates neuronal survival via interacting with TrkB. However, TrkB exists in two different forms: full-length and truncated. TrkB FL is the high-affinity receptor for BDNF and is widely expressed in different regions in the adult brain, for example, hippocampus, striatum, and cortex (Huang and Reichardt, 2003). Under physiological conditions, BDNF binding induces the dimerization of the functional TrkB FL and elicits tyrosine phosphorylation and related cellular neuroprotective signals. In contrast to TrkB FL, TrkB T1 is a predominantly negative isoform. The upregulation of TrkB T1 often indicates the dysfunction of TrkB FL-mediated signals leading to neurodegeneration (Haapasalo et al., 2002; Danelon et al., 2016). Thus, TrkB T1 was suggested as a potential target for the treatment of neurodegenerative diseases (Ferrer et al., 1999; Vidaurre et al., 2012). A recent study reported that the ratio of TrkB FL vs. TrkB T1 was altered in the SNpc and striatum of patients with PD (Fenner et al., 2014). However, EA stimulation was not examined for its effects on the ratio of TrkB FL vs. TrkB T1 in animal models. In the present study, we examined the levels of two TrkB isoforms following MPTP exposure and EA treatment by Western blotting. The key finding from the present study is that EA stimulation can antagonize the effects of MPTP on the formation of TrkB FL and TrkB T1. The neurotoxin MPTP has been well documented for its effects on mitochondrial function and on the overactivation of calpain (Chera et al., 2002). Calpain activation directly enhanced the expression of TrkB T1 (Danelon et al., 2016). These results eventually revealed the mechanisms by which MPTP increases the level of TrkB T1. Nevertheless, EA stimulation restored the balance between TrkB FL and TrkB T1, probably via preventing the MPTP-induced cleavage of TrkB FL or downregulating the TrkB T1 expression. It is well established that TrkB activation initiates the activation of several signaling pathways involving MAPK, ERK, phospholipase Cγ, and PI3K/Akt (Dolcet et al., 1999). Disruption of the TrkB FL/TrkB T1 ratio often alters the cellular signaling transduction. Our result for the phosphorylation of ERK1/2 and Akt also suggested that EA treatment indeed increased the level of phospho-ERK1/2 and phospho-Akt (shown in Figure 3A,B). To further confirm the involvement of the TrkB receptor in EA-induced the neuroprotection, we used the TrkB inhibitor K252a. As expected, K252a completely abrogated the beneficial effect of EA as indicated by behavioral impairments and dopaminergic loss. Moreover, K252a also abolished the effect of EA on the activation of intracellular BDNF/TrkB signal transduction. Together, the current findings suggest that EA-regulated BDNF elevation was accomplished via reversing the imbalance between TrkB FL and TrkB T1.

The present study demonstrated that EA has a protective effect against MPTP-induced neurotoxicity in midbrain dopaminergic neurons and that EA ameliorated the impairments of motor function in a mouse PD model. EA mainly restored the activation of the BDNF-TrkB signaling pathway. Importantly, EA treatment appears to be a promising approach for the management of PD. Considering the general impact of BDNF dysregulation, EA could be a powerful non-pharmacological neuroprotective strategy against PD symptoms. Further work is needed to validate the therapeutic efficacy of EA and elucidate its neuroprotective mechanisms against PD.

## Ethics Statement

All the experimental procedures were performed according to the regulation of the Committee on the Use of Live Animal in Teaching and Research (CULATR No. 4196-16).

## Author Contributions

YZ, DL, and ZN performed the animal experiments. YZ performed the biological experiments, statistical analysis, and wrote the first draft of the manuscript. LL and JR contributed to the design of the study, coordinated the whole process of the study. All the authors contributed to the manuscript revision and approved the final version.

## Conflict of Interest Statement

The authors declare that the research was conducted in the absence of any commercial or financial relationships that could be construed as a potential conflict of interest.
